# Survival benefit with checkpoint inhibitors versus chemotherapy is modified by brain metastases in patients with recurrent small cell lung cancer

**DOI:** 10.3389/fonc.2023.1273478

**Published:** 2023-09-22

**Authors:** Friederike C. Althoff, Lisa V. Schäfer, Fabian Acker, Lukas Aguinarte, Sophie Heinzen, Maximilian Rost, Akin Atmaca, Vivian Rosery, Jürgen Alt, Cornelius F. Waller, Niels Reinmuth, Gernot Rohde, Felix C. Saalfeld, Aaron Becker von Rose, Miriam Möller, Nikolaj Frost, Martin Sebastian, Jan A. Stratmann

**Affiliations:** ^1^ Department of Internal Medicine II, Hematology, Oncology, University Hospital Frankfurt, Frankfurt, Germany; ^2^ Department of Oncology and Hematology, Krankenhaus Nordwest, University Cancer Center Frankfurt (UCT)-University Cancer Center, Frankfurt, Germany; ^3^ Department of Medical Oncology, West German Cancer Center, University Medicine Essen, Essen, Germany; ^4^ Department of Internal Medicine III, Hematology, Oncology, University Medical Center Mainz, Mainz, Germany; ^5^ Department of Internal Medicine I, Haematology, Oncology and Stem Cell Transplantation, Freiburg University Medical Center and Faculty of Medicine, Freiburg, Germany; ^6^ Department of Oncology, Asklepios Clinic München-Gauting, Gauting, Germany; ^7^ Department of Respiratory Medicine, Medical Clinik 1, University Hospital Frankfurt, Frankfurt, Germany; ^8^ Department for Internal Medicine I, University Hospital Carl Gustav Carus Dresden, Technical University of Munich (TU) Dresden, Dresden, Germany; ^9^ Department of Internal Medicine III, Klinikum rechts der Isar, Technical University Munich, Munich, Germany; ^10^ Department of Internal Medicine II, Martha - Maria Hospital Halle, Halle, Germany; ^11^ Charité – Universitätsmedizin Berlin, Corporate Member of Freie Universität Berlin, Humboldt-Universität zu Berlin, and Berlin Institute of Health, Department of Infectious Diseases and Pulmonary Medicine, Berlin, Germany

**Keywords:** small cell lung cancer, recurrent disease, metastatic disease, brain metastases, systemic treatment, checkpoint inhibitors, brain irradiation

## Abstract

**Introduction:**

Small cell lung cancer (SCLC) is a rapidly growing malignancy with early distant metastases. Up to 70% will develop brain metastases, and the poor prognosis of these patients has not changed considerably. The potential of checkpoint inhibitors (CPI) in treating recurrent (r/r) SCLC and their effect on brain metastases remain unclear.

**Methods:**

In this retrospective multicenter study, we analyzed r/r SCLC patients receiving second or further-line CPI versus chemotherapy between 2010 and 2020. We applied multivariable-adjusted Cox regression analysis to test for differences in 1-year mortality and real-world progression. We then used interaction analysis to evaluate whether brain metastases (BM) and/or cranial radiotherapy (CRT) modified the effect of CPI versus chemotherapy on overall survival.

**Results:**

Among 285 patients, 99 (35%) received CPI and 186 (65%) patients received chemotherapy. Most patients (93%) in the CPI group received nivolumab/ipilimumab. Chemotherapy patients were entirely CPI-naïve and only one CPI patient had received atezolizumab for first-line treatment. CPI was associated with a lower risk of 1-year mortality (adjusted Hazard Ratio [HR_adj_] 0.59, 95% CI 0.42 to 0.82, p=0.002). This benefit was modified by BM and CRT, indicating a pronounced effect in patients without BM (with CRT: HR_adj_ 0.34, p=0.003; no CRT: HR_adj_ 0.50, p=0.05), while there was no effect in patients with BM who received CRT (HR_adj_ 0.85, p=0.59).

**Conclusion:**

CPI was associated with a lower risk of 1-year mortality compared to chemotherapy. However, the effect on OS was significantly modified by intracranial disease and radiotherapy, suggesting the benefit was driven by patients without BM.

## 1 Introduction

Small cell lung cancer (SCLC) is an aggressive and rapidly growing malignancy with early metastases. Among the 70% of patients presenting with extensive disease at initial diagnosis, the 5-year overall survival remains less than 5% (1). While response rates to first-line platinum plus etoposide chemotherapy are as high as 60 to 70% in patients with extensive disease, data have demonstrated early disease recurrence (2). For refractory or relapsed (r/r) SCLC, treatment options are scarce (3).

Over the last decade, clinical trials have evaluated a variety of novel agents for the treatment of SCLC. In the IMpower-133 and CASPIAN phase 3 trials, the addition of checkpoint inhibitors (CPI) to platinum-based chemotherapy modestly improved overall survival (OS), leading to an approval of atezolizumab and durvalumab for first-line treatment in combination with platinum and etoposide (4, 5). Moreover, the Chinese phase 3 trials ASTRUM and CAPSTONE-1 have confirmed an OS benefit by adding the CPI serplulimab and adebrelimab, respectively, to first-line platinum/etoposide (6, 7). For patients with r/r SCLC, further trials such as CheckMate032, KeyNote158, and KeyNote028 evaluated the use of CPI in second or further-line treatment regimen (8–10). In this pre-treated, CPI-naïve setting, results have been inconclusive, and a potential survival benefit of CPI over chemotherapy could not be demonstrated. As a consequence, temporary FDA approvals of nivolumab and pembrolizumab for pre-treated SCLC patients were withdrawn in early 2021.

Importantly, every fifth patient presents with brain metastases (BM) at disease onset and an additional 50% will develop BM during the course of their disease (1, 11). BM are particularly challenging due to often detrimental effects on the patient’s performance status and their poor response to systemic agents with limited penetration of the blood-brain barrier, resulting in a significant shorter median OS of 8.5 versus 12.6 months (5). Whole-brain radiotherapy (WBRT) still remains the standard treatment in these patients but is associated with a worsening quality of life and neurocognitive function (12). The potential of CPI in treating patients with r/r SCLC and their effect on BM remain unclear. In an exploratory analysis of the CASPIAN trial, the authors suggested the OS benefit was maintained irrespective of the presence or absence of BM (13).

In this retrospective multicenter cohort study, we hypothesized that treatment with CPI versus chemotherapy improved overall survival and real-world progression-free survival in r/r SCLC. We then evaluated whether brain metastases and/or cranial radiotherapy modified the effect of CPI versus chemotherapy on survival in this hard-to-treat patient population.

## 2 Materials and methods

### 2.1 Study design

We conducted a multicenter, retrospective cohort study to analyze the effect of second or further-line (≥2L) CPI versus chemotherapy on survival in adult patients with r/r SCLC. Patient data were obtained between 2010 and 2020 at 11 academic healthcare institutions across Germany, including university hospitals and specialized treatment centers. Patients were eligible for inclusion if they received treatment for refractory/recurrent, incurable, extensive disease SCLC. We included patients in the CPI group if they were treated with single or double CPI regimen. All patients had received at least one previous non-curative treatment line. Patients who received CPI within a clinical trial were excluded. Since CPI had not been approved by the European Medical Agency for the treatment of r/r SCLC, their therapeutic use was limited to cases where a funding request to cover the costs had been accepted by the health insurance provider. However, due to the limited treatment options available, requests for reimbursement were made on a regular basis as an individual therapeutic trial, as described in detail previously (14). The study was approved by the local ethics committee at the University Hospital Frankfurt, and a data use agreement was established between institutions (protocol number UCT-2-2020). Data were collected from electronic hospital-registry databases and merged into a combined dataset after strict de-identification within the respective hospital network. This manuscript adheres to the STROBE guidelines for reporting observational studies (**Supplemental Digital Content**; **Table S1**) (15).

### 2.2 Primary and secondary analysis

We used a multivariable-adjusted Cox proportional hazards regression to investigate the effect of CPI versus chemotherapy on 1-year OS and real-world progression-free survival (rwPFS), respectively. Analyses started on the first day that the patient received the treatment (day 1 of the first cycle of CPI/chemotherapy) to avoid immortal time bias and ensure that all time intervals during which patients may have experienced the outcome were captured in the analysis. Analyses were adjusted for confounding variables based on literature review and clinical plausibility. Confounding variables included age (quintiles), sex, progressive disease within 180 days of first-line treatment, prior cranial radiotherapy (CRT), a history of brain metastases (BM), and liver metastases. Regarding the tumor staging at the time of this investigation, we present a homogenous cohort of patients with incurable, extensive, stage IV disease as all patients had previously shown tumor progression (r/r SCLC). We provide the UICC tumor staging at the time of initial diagnosis (**Table 1**), albeit this initial staging was considered to have no impact on the outcome of the recurrent disease. We did not assess co-existing malignancies as the SCLC and its metastases were judged as the major determinants of the prognosis even when multiple cancers exist. We tested for violation of the proportional hazards assumption and utilized the Cox regression model to estimate hazard ratios with 95% confidence intervals. Additionally, we performed univariate Kaplan-Meier analysis using logrank-test.

**Table 1:** Baseline patient characteristics of the full study cohort across treatment groups.

Variables	Chemotherapy N=186 (65%)	Checkpoint inhibitor N=99 (35%)	P-value
Age (y), mean ± SD	62.6 ± 8.6	61.0 ± 9.3	0.16
Sex, female, n (%)	63 (33.9%)	37 (37.4%)	0.56
Smoking, n (%)			<0.001
Never smoker	2 (1.1%)	3 (3.0%)	
Smoker	77 (41.4%)	40 (40.4%)	
Ex-smoker	56 (30.1%)	47 (47.5%)	
n.a.	51 (27.4%)	9 (9.1%)	
Pack years, median (IQR)	40 (30, 50)	31 (20, 42)	0.009
Pathology, n (%)			0.37
SCLC	179 (96.2%)	95 (96.0%)	
LCNEC	7 (3.8%)	3 (3.0%)	
Not otherwise specified (nos)	0 (0%)	1 (1.0%)	
UICC staging at the time of initial diagnosis, n (%)			<0.001
IA	0 (0%)	1 (1.0%)	
IB	0 (0%)	2 (1.7%)	
IIA	2 (1.1%)	1 (1.0%)	
IIB	0 (0%)	1 (1.0%)	
III, nos	0 (0%)	3 (3.0%)	
IIIA	5 (2.7%)	13 (13.1%)	
IIIB	2 (1.1%)	9 (9.1%)	
IIIC	1 (0.5%)	5 (5.1%)	
IV, nos	147 (79.0%)	27 (27.3%)	
IVA	7 (3.8%)	10 (10.1%)	
IVB	22 (11.8%)	26 (22.3%)	
Extensive disease at the time of initial diagnosis, n (%)	175 (94.1%)	63 (63.6%)	<0.001
Drugs of first-line (1L), n (%)			0.008
Cisplatin/etoposide	108 (58.1%)	42 (42.4%)	
Carboplatin/etoposide	73 (39.2%)	56 (56.6%)	
Carboplatin/etoposide/atezolizumab	0 (0%)	1 (1%)	
n.a.	5 (2.7%)	0 (0%)	
Brain imaging prior to start of 1L, n (%)			<0.001
No brain imaging	65 (34.9%)	33 (33.3%)	
MRI	88 (47.3%)	64 (64.6%)	
CT	33 (17.7%)	2 (2.0%)	
Best response to 1L treatment, n (%)			<0.001
CR	2 (1.3%)	3 (3.4%)	
PR	16 (10.5%)	15 (16.9%)	
SD	52 (34.2%)	9 (10.1%)	
PD	82 (53.9%)	62 (69.7%)	
n.a.	34 (18.3%)	10 (10.1%)	
Progression on 1L within 365 days, n (%)	128 (68.8%)	75 (75.8%)	0.22
Progression on 1L within 180 days, n (%)	62 (33.5%)	32 (32.3%)	0.57
ECOG at start of ≥2L treatment, median (IQR)	1 (0, 1)	1 (1, 2)	0.10
Metastases at start of ≥2L treatment, n (%)			
Lung	26 (14%)	47 (47.5%)	<0.001
Liver	80 (43%)	43 (43.4%)	0.95
Adrenal glands	45 (24.2%)	23 (23.2%)	0.86
Bone	64 (34.4%)	25 (25.3%)	0.11
Brain	75 (40.3%)	41 (41.4%)	0.86
CNS category at start of ≥2L treatment, n (%)			0.79
No CRT, no BM	52 (28%)	30 (30.3%)	
No CRT, with BM	12 (6.5%)	9 (9.1%)	
With CRT, no BM	59 (31.7%)	28 (28.3%)	
With CRT, with BM	63 (33.9%)	32 (32.3%)	
Drugs of ≥2L treatment, n (%)			<0.001
Topotecan	110 (59.1%)	0 (0%)	
Carboplatin/etoposide	43 (23.1%)	0 (0%)	
Cisplatin/etoposide	10 (5.4%)	0 (0%)	
Adriamycin/cyclophosphamide/vincristin	12 (6.5%)	0 (0%)	
Epirubicin/cyclophosphamide/vincristin	4 (2.2%)	0 (0%)	
Docetaxel	2 (1.1%)	0 (0%)	
Mitomycin/gemcitabine/cisplatin	1 (0.5%)	0 (0%)	
Etoposide	1 (0.5%)	0 (0%)	
Alisertib/paclitaxel	1 (0.5%)	0 (0%)	
Other	1 (0.5%)	0 (0%)	
Nivolumab/ipilimumab	0 (0%)	92 (93.0%)	
Nivolumab	0 (0%)	7 (7.0%)	

BM, brain metastases; CPI, checkpoint inhibitor; CR, complete remission; CRT, cranial radiotherapy; ECOG, Eastern Cooperative Oncology Group; LCNEC, large cell neuroendocrine carcinoma; n.a., not available; nos, not otherwise specified; PD, progressive disease; PR, partial remission; SCLC, small cell lung cancer; SD, stable disease; 1L, first line of treatment; ≥2L, second or further-line treatment.

The UICC tumor staging refers to the time of initial diagnosis, while all included patients had r/r SCLC with incurable, extensive, stage IV disease at the time of this study.

### 2.3 Effect modification by brain metastases and/or cranial radiotherapy

To investigate whether the effect of CPI versus chemotherapy on 1-year OS was modified by a patient’s history of BM and/or cranial radiotherapy (CRT), we included an interaction term between the primary exposure and the individual patient’s “CNS category” in the Cox regression model. For the interaction term “CNS category”, patients were divided into eight groups by ≥2L treatment (CPI versus chemotherapy), BM (binary), and CRT (binary). Interaction analyses were performed across groups of the interaction term. Comparisons were made with the baseline group of patients who received chemotherapy, had no history of BM, and did not receive CRT. Subgroups are displayed in **Table 1**.

In addition, we analyzed a subgroup of patients where brain imaging was available within six weeks before treatment initiation to provide further information on intracranial progression.

### 2.4 Sensitivity analyses

In sensitivity analyses, we used a multivariable-adjusted standard logistic regression and marginal effects to estimate the adjusted risk of 1-year mortality per 100 patients across groups. In addition, we applied propensity score analyses to address the possibility of unbalanced confounding between patients receiving CPI versus chemotherapy for ≥2L treatment. Both inverse probability of treatment weighting and 1:1 propensity score matching was used to assess the robustness of the primary association to analytic approach. The propensity score for a patient was defined as the probability of receiving CPI versus chemotherapy, conditional on all covariates described for the primary analysis. Based on the estimated propensity score, patients were matched on a 1:1 basis using an algorithm with a caliper of 0.1 without replacement (16). This algorithm identifies matched pairs within a closeness range of 0.00001 of the propensity score. Only if no more patients are identified for matching, the program then selects pairs in a range of 0.0001, 0.001, 0.01, up to a range of 0.1. Variables were examined for residual imbalances. Matching effectiveness was evaluated by calculating standardized differences of confounding variables after propensity score adjustment. In the propensity score matched cohort, we used a logistic regression model on the primary outcome and included confounding variables with a standardized difference of more than 0.1 (17, 18). Additionally, we performed Cox regression and Kaplan-Meier survival analyses in the matched cohort. Moreover, we used propensity score estimates in an inverse probability of treatment weighting model (19). We further included additional confounding variables into the primary model such as best response to first line treatment (complete response (CR), partial response (PR), stable disease (SD), progressive disease (PD)), the Eastern Cooperative Oncology Group (ECOG) performance status at start of ≥2L treatment, and the UICC staging at the time of initial diagnosis, respectively, to test for robustness of the primary analysis. Finally, we provide data on three-year survival along with a “number at risk”-table using Kaplan Meier analysis.

### 2.5 Statistical analyses

The primary outcome was 1-year overall survival (OS) following initiation of ≥2L treatment with CPI versus chemotherapy. The secondary outcome was 1-year rwPFS. Tumor response assessments were obtained in clinical routine and were performed without an independent review. The assumption of linearity between the outcome and continuous covariates was tested using scatter plots (**Supplemental Digital Content**; **Figure S2**). To adjust for non-linear relationships, continuous confounding variables were divided into quintiles. Cases with missing data required for statistical analyses were excluded using the complete-case approach. A two-sided p-value of <0.05 was considered statistically significant. Data analyses were performed using Stata (StataCorp LP, version 13.0).

## 3 Results

### 3.1 Study cohort

**Figure 1:**
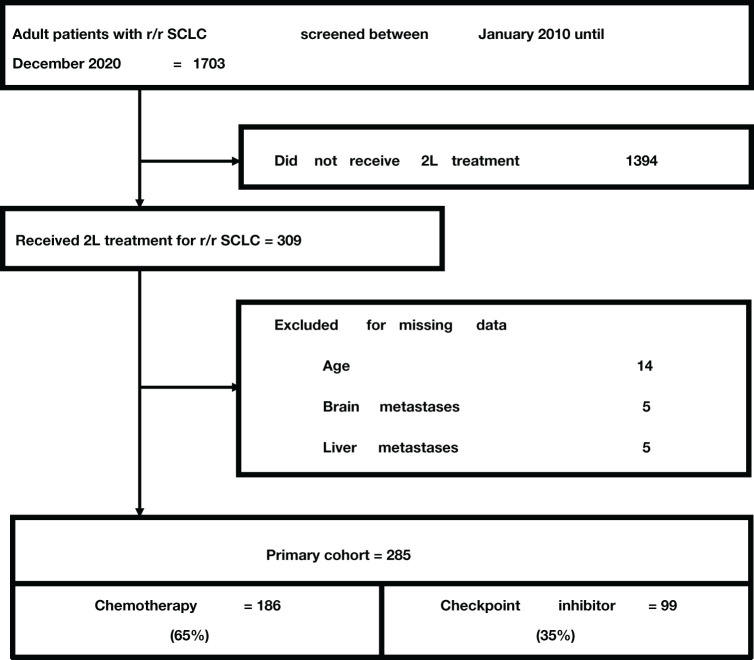
Study flow chart.

In total, 1703 patients with r/r SCLC were considered for inclusion, of which only 309 (18%) patients received second-line treatment. After application of the exclusion criteria, the final cohort consisted of 285 patients (**Figure 1**). 186 (65%) patients received chemotherapy and 99 (35%) received CPI for second or further-line treatment (≥2L) of r/r SCLC. In the CPI group, 26% of patients received CPI as second-line, 46% as third-line, 16% as fourth-line, 11% as fifth-line, and 1% as sixth-line treatment. The median number of prior treatment lines was 2 (IQR 1 to 3). No patient in the chemotherapy group had previously received a checkpoint inhibitor, while one patient in the CPI group had received atezolizumab in combination with carboplatin and etoposide for first-line treatment. Most patients in the CPI group received double-CPI, combining the PD-1 inhibitor nivolumab with the CTLA-4 inhibitor ipilimumab in 93% of cases, some patients received nivolumab monotherapy (7%). Treatment with chemotherapy most often included topotecan in 59.1% of patients, followed by carboplatin or cisplatin plus etoposide in 23.1% and 5.4%, respectively, ACO (adriamycin, cyclophosphamide, and vincristine) in 6.5%, and other chemotherapy regimen (**Table 1**). Patient characteristics and distribution of confounding variables by treatment groups are provided in **Table 1**. The median follow-up time was 30.8 months (95% confidence interval (CI) 23.3 to 38.3 months) according to the method provided by Schemper & Smith (20). The total range was 1 to 1404 days.

### 3.2 One-year overall survival

**Figure 2:**
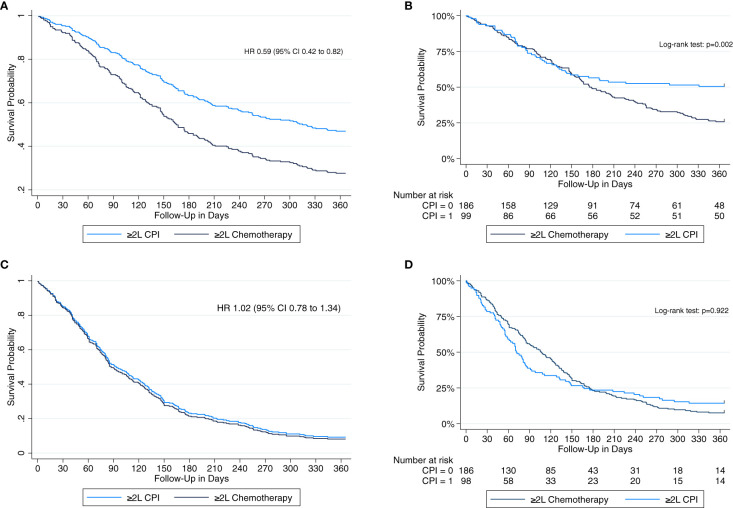
Multivariable-adjusted cox proportional hazards regression to estimate hazard ratios (HR) with 95% confidence intervals (CI) and univariate Kaplan-Meier survival analysis including a ‘number at risk’ table and a logrank test to analyze 1-year overall survival **(A, B)** and 1-year real-world progression-free survival **(C, D)**, respectively, among patients receiving second or further-line (≥2L) treatment with checkpoint inhibitors (CPI) versus chemotherapy.

In total, 187 (65.6%) patients died within one year of initiation of ≥2L treatment, 138 (74.2%) with chemotherapy and 49 (49.5%) with CPI. The median OS was 6.3 months (95% confidence interval (CI) 5.4 to 7.9). CPI versus chemotherapy was associated with an improved 1-year overall survival in unadjusted (HR 0.60, 95% CI 0.44 to 0.84, p=0.003) as well as adjusted analyses (adjusted HR [HR_adj_] 0.59, 95% CI 0.42 to 0.82, p=0.002). Survival curves from Kaplan Meier estimates (Log-rank test p=0.002) and Cox regression analysis are shown in **Figures 2A, B**. In the primary confounder model, brain metastases (HR 1.92, 95% CI 1.36 to 2.69, p<0.001) and liver metastases (HR 1.40, 95% CI 1.04 to 1.90, p=0.026) were independent risk factors of mortality, respectively, while a prior cranial radiotherapy was associated with a lower risk (HR 0.66, 95% CI 0.47 to 0.94, p=0.021).

### 3.3 One-year real-world progression-free survival

The median rwPFS was 2.9 months (95% CI 2.6 to 3.6). There was no difference in 1-year rwPFS between patients receiving CPI versus chemotherapy in unadjusted (HR 1.01, 95% CI 0.78 to 1.32, p=0.922) and adjusted analyses (HR_adj_ 1.02, 95% CI 0.78 to 1.34, p=0.884). Cox regression and Kaplan-Meier survival curves are displayed in **Figures 2C, D**.

### 3.4 Analyses on the role of brain metastases and/or cranial radiotherapy

The effect of CPI versus chemotherapy on 1-year OS was significantly modified by a patient’s history of BM and/or prior CRT towards a more pronounced effect among patients without brain metastases. The strongest effect was observed in patients without BM who received CPI and a prior CRT (HR_adj_ 0.34, 95% CI 0.17 to 0.69, p=0.003), followed by patients without BM who received CPI but no prior CRT (HR_adj_ 0.50, 95% CI 0.25 to 0.99, p=0.05). Results suggested a trend for an OS improvement in patients with BM who received CPI and a prior CRT (HR_adj_ 0.85, 95% CI 0.47 to 1.54, p=0.59), however, there was no significant difference compared to baseline. In a small sub-cohort of only nine patients with BM who received CPI but no CRT, OS was significantly worse compared to baseline (HR_adj_ 2.89, 95% CI 1.20 to 6.98, p=0.02, **Table 2**).

**Table 2:** Results of interaction analysis demonstrating a modification of the primary effect of CPI versus chemotherapy on 1-year OS by a patient’s history of brain metastases and/or CRT.

Subgroup	N of pts.	HR (95% CI)	P-value
*Baseline=CT, no CRT, no BM*	52	-	-
CT, no CRT, with BM	12	1.77 (0.88-3.56)	0.109
CT, with CRT, no BM	59	0.68 (0.43-1.08)	0.104
CT, with CRT, with BM	63	1.12 (0.73-1.73)	0.601
CPI, no CRT, no BM	30	0.50 (0.25-0.99)	0.05
CPI, no CRT, with BM	9	2.89 (1.20-6.98)	0.018
CPI, with CRT, no BM	28	0.34 (0.17-0.69)	0.003
CPI, with CRT, with BM	32	0.85 (0.47-1.54)	0.602

The aRD is the absolute risk difference in the observed risk between the two groups. The negative value (minus symbol) means that CPI were associated with a decreased risk of 1y-mortality (by 26%).

In a subgroup of 79 patients where brain imaging was available within six weeks before treatment initiation, 16/26 (64%) patients who received CPI and 52/53 (98%) patients who received chemotherapy had brain metastases. Whole brain irradiation had already been performed in 21/26 (81%) and 48/53 (91%) patients who received CPI versus chemotherapy, respectively. The median (IQR) time to intracranial real-world progression was 71 (31, 144) days after initiation of CPI and 97 (71, 172) days after chemotherapy. There was no difference in 1-year intracranial rwPFS (HR 1.49, 95% CI 0.78 to 2.86, p=0.226), adjusting for prior brain irradiation.

### 3.5 Sensitivity analyses

In standard logistic regression analysis, 1-year mortality was significantly lower in patients receiving CPI versus chemotherapy (OR_adj_ 0.31, 95% CI 0.18 to 0.53, p<0.001; **Table 3**). The adjusted risk of 1-year mortality was 74 deaths (95% CI 69 to 82) per 100 patients treated with chemotherapy and 49 deaths (95% CI 39 to 59) per 100 patients treated with CPI (p<0.001). In the 1:1 propensity-score matched cohort including 198 patients, we compared 99 patients receiving chemotherapy with 99 patients receiving CPI. Patient characteristics in the propensity score matched cohort were well balanced between treatment groups and are provided in the **Supplemental Digital Content**; **Table S2**. Propensity score matching confirmed a lower risk of 1-year mortality in patients with CPI versus chemotherapy (OR_adj_ 0.32, 95% CI 0.17 to 0.58, p<0.001), with an adjusted absolute risk difference of -25.9% (95% CI -39% to -13%, p=0.0003; **Table 3**). The median OS in the PSM cohort was 6.5 months (95% CI 5.5 to 8.4). When applying the Cox model to the PSM cohort, CPI versus chemotherapy was associated with an improved 1-year OS (HR 0.61, 95% CI 0.44 to 0.88, p=0.008; **Table 3**). Kaplan Meier estimates using logrank testing confirmed a significant difference (p=0.0072). Following inverse probability of treatment weighting with confounders of the primary analysis, treatment with CPI was significantly associated with a lower risk of 1-year mortality (OR_adj_ 0.24, 95%CI 0.20 to 0.27, p<0.001; **Table 3**). Results were robust when including additional confounding variables into the primary Cox regression model (**Table 3**). Kaplan Meier estimates on three-year OS confirmed a significant benefit in the CPI group (p<0.001) and are provided in **Figure S1** in the **Supplemental Digital Content**.

**Table 3:** Primary outcome across analyses.

Analysis	N of pts.	Effect measure (95% CI)	P-value
Cox proportional hazards regression (HR)	285	0.59 (0.42 to 0.82)	=0.002
Standard logistic regression (OR)	285	0.31 (0.18 to 0.53)	<0.001
Inverse probability weighting (OR)	285	0.24 (0.20 to 0.27)	<0.001
Propensity score matching (aRD)	198	-25.9% (-39% to -13%)	=0.0003
Cox proportional hazards regression in the PSM cohort (HR)	198	0.61 (0.44 to 0.88)	=0.008
Additional confounding variables (HR):			
Best response to 1L treatment	258	0.60 (0.41 to 0.89)	=0.011
ECOG at start of ≥2L treatment	235	0.65 (0.45 to 0.93)	=0.018
UICC staging at the time of initial diagnosis (UICC IB to IIIB versus IIIC to IVB)	281	0.60 (0.42 to 0.86)	=0.006

Association between ≥2L treatment with CPI versus chemotherapy and 1-year overall survival obtained from multivariable-adjusted Cox proportional hazards regression, standard logistic regression, inverse probability of treatment weighting, propensity score matching (PSM) analysis, and when including additional confounding variables into the primary Cox regression model.

## 4 Discussion

In this retrospective real-world multicenter study of more than 280 patients with refractory or recurrent small cell lung cancer, second or further-line treatment with checkpoint inhibitors was associated with a lower risk of 1-year mortality compared with chemotherapy. However, the effect on overall survival was significantly modified by a patient’s history of brain metastases and/or cranial radiotherapy. The benefit was magnified in patients without brain metastases (with or without radiotherapy), while there was no difference between CPI and chemotherapy in patients with brain metastases who received radiotherapy. Our data suggest the overall survival benefit with CPI was driven by patients without brain metastases.

In line with epidemiological data, our real-world cohort of pre-treated patients included 40.7% with brain metastases and 64% of all patients had received brain irradiation. In the CASPIAN trial on the use of first-line durvalumab plus carboplatin/etoposide (4), 10.2% of patients had BM that were asymptomatic or treated and stable, and 23% of all patients received radiotherapy to the brain. Of note, 90% of those patients with BM had not received a prior brain radiation at study entry. The authors performed *post-hoc* subgroup analyses to evaluate the role of intracranial disease, and concluded an improved overall survival by the addition of CPI was irrespective of whether or not patients presented with BM (13). However, in the CASPIAN subgroups, a potential trend towards an improved OS did not reach significance among patients with BM (HR 0.79, 95% CI 0.44 to 1.41), while there was a clear benefit among those without BM (HR 0.76, 95% CI 0.62 to 0.92). In the present study, we performed an interaction analysis to test for significant differences across groups. Our data demonstrated that the effect of CPI versus chemotherapy on OS was significantly modified by a patient’s history of BM and cranial radiotherapy, indicating a pronounced benefit of CPI among patients without BM, while there was no difference between CPI versus chemotherapy in patients with prior BM who received CRT. Similarly, when looking at data from the first-line setting, the IMpower133-study and the KeyNote604-study demonstrated that patients with BM did not benefit from the addition of atezolizumab or pembrolizumab, respectively, to standard chemotherapy with platinum and etoposide (5, 21).

Overall, there is limited evidence on the intracerebral efficacy of CPI-based therapies as the majority of trials included only patients with asymptomatic or treated brain metastases. Among non-small cell lung cancer (NSCLC) patients, intracranial response rates were high when treated with first-line combined chemoimmunotherapy, such as camrelizumab with carboplatin/pemetrexed from the CAP-BRAIN trial (22) (intracranial ORR 46.7%) and atezolizumab with carboplatin/pemetrexed from the ATEZO-BRAIN trial (intracranial ORR 40%) (23). In contrast, intracranial response was lower in a phase II trial that used a single-CPI regimen (29.7% with pembrolizumab monotherapy) in patients with or without previous systemic treatment but naïve to PD-1 and PD-L1 inhibitors (24). In NSCLC, discussions have started more recently as to whether radiotherapy to the brain (especially whole brain radiotherapy, WBRT) can be initially omitted in some patients to reduce the associated risk of neurocognitive deterioration, while still maintaining local tumor control by improved systemic treatment options. In SCLC, from our perspective irradiation remains the important standard of care to treat brain metastases in every patient, including WBRT and stereotactic radiosurgery where possible. Our data indicate superior survival in patients who received brain radiation, irrespective of whether CPI or chemotherapy were used as systemic treatment, which is also acknowledged by current guidelines (1, 25).

The presentation of real-world SCLC patients not selected by strict inclusion criteria may provide a more generalisable conclusion on high-risk subgroups such as patients with brain metastases. Nevertheless, this study was retrospective in nature with several limitations. Tumor response assessments were obtained in clinical routine not following standardized criteria and without an independent review, which may limit direct comparability to prospective trials. For instance, unpublished data from the real-world, prospective, multicenter clinical research platform into molecular testing, treatment, and outcome data on lung carcinoma patients (CRISP) in Germany between 2019 and 2021 suggest higher rates of patients receiving second-line treatment (40%) for stage IV SCLC, while 31% died prior to second-line treatment. In contrast to our patients, 73% of that more current cohort received chemotherapy with CPI for first-line treatment. One concern in the present study is that patients who received a chemotherapy-based treatment may have had a higher need of a fast remission-induction than those who received CPI. The two groups were similar with respect to metastatic sites that were independent risk factors of mortality, such as brain (40.3 versus 41.4%) and liver (43 versus 43.3%). More patients in the chemotherapy group had extensive disease at the time of disease onset (94 versus 64%). However, rates of primary progressive disease as best response upon first-line chemotherapy were higher in the CPI group (11 versus 30%). Predominant use of the doublet of nivolumab/ipilimumab in the CPI-group may indicate a good and perhaps better general condition in these patients. Importantly, the improved overall survival with CPI versus chemotherapy remained robust across several sensitivity analyses. We conducted a propensity score matching as well as an inverse probability weighting, both statistical methods that have been designed to partly reduce bias due to unbalanced confounding. Patient characteristics across groups were well-balanced after using propensity score matching, and both statistical approaches yielded similar results, which strengthened our confidence in the finding. However, these methods can only address bias due to measured covariates, while residual unmeasured confounding also exists. To inform decision-making in the treatment choice for r/r SCLC especially for patients with BM, future research on the effectiveness of CPI should investigate differences by molecular subtypes of SCLC, ideally in a prospective setting. Further tumor-related factors predictive of the effectiveness of CPI in SCLC are still largely unknown (14, 26).

Finally, our real-world study in recurrent SCLC only partly supports the primary hypothesis that CPI compared with chemotherapy are associated with an improved overall survival. Interaction analysis revealed that this benefit was driven by patients without brain metastases, while no difference could be observed among patients with BM. Patients with BM represent a risk group where CPI do not seem to add any benefit to standard chemotherapy and may even bring additional risks. This may also be true in the first-line setting, where *post-hoc* subgroup analyses of clinical trials did not demonstrate a survival benefit by the addition of CPI in patients with BM. Instead of the current practice of treating all SCLC patients with first-line combined chemoimmunotherapy, it would be clinically relevant to identify whether certain subsets of patients with BM do benefit from CPI. Only a prospective randomized comparison between chemoimmunotherapy and chemotherapy for first-line treatment of patients with SCLC and BM could answer this question. Importantly, future studies of newer agents such as antibody drug conjugates and bispecific T-cell engagers targeting DLL3 as well as inhibitors of EHZ2 or PARP should evaluate the intracranial efficacy to finally address the unmet need in SCLC patients with brain metastases.

## Data availability statement

The raw data supporting the conclusions of this article will be made available by the authors, without undue reservation.

## Ethics statement

The studies involving humans were approved by The Ethics Committee at the University Hospital Frankfurt. The studies were conducted in accordance with the local legislation and institutional requirements. The ethics committee/institutional review board waived the requirement of written informed consent for participation from the participants or the participants’ legal guardians/next of kin because of the retrospective study design using de-identified data.

## Author contributions

FCA: Conceptualization, Data curation, Formal Analysis, Investigation, Methodology, Project administration, Software, Validation, Visualization, Writing – original draft. LS: Writing – review & editing, Conceptualization, Data curation, Investigation, Project administration, Visualization. FA: Methodology, Validation, Visualization, Writing – review & editing. LA: Methodology, Validation, Visualization, Writing – review & editing. SH: Methodology, Validation, Visualization, Writing – review & editing. MR: Methodology, Validation, Visualization, Writing – review & editing. AA: Data curation, Project administration, Resources, Validation, Writing – review & editing. VR: Data curation, Project administration, Resources, Writing – review & editing. JA: Data curation, Project administration, Resources, Validation, Writing – review & editing. CW: Data curation, Project administration, Resources, Validation, Writing – review & editing. NR: Data curation, Project administration, Resources, Validation, Writing – review & editing. GR: Data curation, Project administration, Resources, Validation, Writing – review & editing. FS: Data curation, Project administration, Resources, Validation, Writing – review & editing. AB: Data curation, Project administration, Resources, Validation, Writing – review & editing. MM: Data curation, Project administration, Resources, Validation, Writing – review & editing. NF: Data curation, Project administration, Resources, Validation, Writing – review & editing. MS: Conceptualization, Data curation, Formal Analysis, Investigation, Methodology, Project administration, Resources, Supervision, Validation, Visualization, Writing – review & editing. JAS: Data curation, Formal Analysis, Investigation, Methodology, Project administration, Resources, Supervision, Validation, Visualization, Writing – review & editing.
