# Supplementary material for: Survival benefit with checkpoint inhibitors versus chemotherapy is modified by brain metastases in patients with recurrent small cell lung cancer
Source: Front Oncol. 2023 Sep 22;13:1273478. doi: 10.3389/fonc.2023.1273478 (PMC10556470; doi:10.3389/fonc.2023.1273478)
Supplement: Supplementary file 1 [file DataSheet_1.docx]

Supplementary Material

Survival benefit with checkpoint inhibitors versus chemotherapy is modified by brain metastases in patients with recurrent small cell lung cancer

**Friederike C. Althoff^*^, Lisa V. Schäfer, Fabian Acker, Lukas Aguinarte, Sophie Heinzen, Maximilian Rost, Akin Atmaca, Vivian Rosery, Jürgen Alt, Cornelius F. Waller, Niels Reinmuth, Gernot Rohde, Felix Saalfeld, Aaron Becker von Rose, Miriam Möller, Nikolaj Frost, Martin Sebastian, Jan A. Stratmann**

*** Correspondence:**Friederike C. Althoff
[friederike.althoff@kgu.de](mailto:friederike.althoff@kgu.de)

# Supplementary Tables

## Table S1. STROBE statement: Checklist of items that should be included in reports of observational studies (E. von Elm, D.G. Altman, M. Egger, S.J. Pocock, P.C. Gøtzsche, J.P. Vandenbroucke, The Strengthening the Reporting of Observational Studies in Epidemiology (STROBE) statement: guidelines for reporting observational studies, J. Clin. Epidemiol. 61 (2008) 344–349. <https://doi.org/10.1016/j.jclinepi.2007.11.008>).

| **Section/Topic** | Item No | Recommendation | Reported on Page No |
| --- | --- | --- | --- |
| **Title and abstract** | 1 | (*a*) Indicate the study’s design with a commonly used term in the title or the abstract | 1-2 |
|  |  | (*b*) Provide in the abstract an informative and balanced summary of what was done and what was found | 1-2 |
| Introduction | | | |
| Background/rationale | 2 | Explain the scientific background and rationale for the investigation being reported | 2 |
| Objectives | 3 | State specific objectives, including any prespecified hypotheses | 2-3 |
| Methods | | | |
| Study design | 4 | Present key elements of study design early in the paper | 3 |
| Setting | 5 | Describe the setting, locations, and relevant dates, including periods of recruitment, exposure, follow-up, and data collection | 3 |
| Participants | 6 | (*a*) *Cohort study*—Give the eligibility criteria, and the sources and methods of selection of participants. Describe methods of follow-up  *Case-control study*—Give the eligibility criteria, and the sources and methods of case ascertainment and control selection. Give the rationale for the choice of cases and controls  *Cross-sectional study*—Give the eligibility criteria, and the sources and methods of selection of participants | 3 |
|  |  | (*b*) *Cohort study*—For matched studies, give matching criteria and number of exposed and unexposed  *Case-control study*—For matched studies, give matching criteria and the number of controls per case | 3-4 |
| Variables | 7 | Clearly define all outcomes, exposures, predictors, potential confounders, and effect modifiers. Give diagnostic criteria, if applicable | 3-4 |
| Data sources/measurement | 8* | For each variable of interest, give sources of data and details of methods of assessment (measurement). Describe comparability of assessment methods if there is more than one group | 3-4 |
| Bias | 9 | Describe any efforts to address potential sources of bias | 3-4 |
| Study size | 10 | Explain how the study size was arrived at | 3, Fig 1 |
| Quantitative variables | 11 | Explain how quantitative variables were handled in the analyses. If applicable, describe which groupings were chosen and why | 3 |
| Statistical methods | 12 | (*a*) Describe all statistical methods, including those used to control for confounding | 3-4 |
|  |  | (*b*) Describe any methods used to examine subgroups and interactions | 3-4 |
|  |  | (*c*) Explain how missing data were addressed | 3-4 |
|  |  | (*d*) *Cohort study*—If applicable, explain how loss to follow-up was addressed  *Case-control study*—If applicable, explain how matching of cases and controls was addressed  *Cross-sectional study*—If applicable, describe analytical methods taking account of sampling strategy | 3 |
|  |  | (*e*) Describe any sensitivity analyses | 4 |

| Results | | | |
| --- | --- | --- | --- |
| Participants | 13* | (a) Report numbers of individuals at each stage of study—eg numbers potentially eligible, examined for eligibility, confirmed eligible, included in the study, completing follow-up, and analysed | 4-5, Fig 1 |
|  |  | (b) Give reasons for non-participation at each stage | 4-5, Fig 1 |
|  |  | (c) Consider use of a flow diagram | Fig 1 |
| Descriptive data | 14* | (a) Give characteristics of study participants (eg demographic, clinical, social) and information on exposures and potential confounders | 4-5, Tab 1 |
|  |  | (b) Indicate number of participants with missing data for each variable of interest | Fig 1, Tab 1 |
|  |  | (c) *Cohort study*—Summarise follow-up time (eg, average and total amount) | 5 |
| Outcome data | 15* | *Cohort study*—Report numbers of outcome events or summary measures over time | 5, Tab 1 |
|  |  | *Case-control study—*Report numbers in each exposure category, or summary measures of exposure |  |
|  |  | *Cross-sectional study—*Report numbers of outcome events or summary measures |  |
| Main results | 16 | (*a*) Give unadjusted estimates and, if applicable, confounder-adjusted estimates and their precision (eg, 95% confidence interval). Make clear which confounders were adjusted for and why they were included | 4-5, Tab 2, Tab 3 |
|  |  | (*b*) Report category boundaries when continuous variables were categorized | 4-5 |
|  |  | (*c*) If relevant, consider translating estimates of relative risk into absolute risk for a meaningful time period |  |
| Other analyses | 17 | Report other analyses done—eg analyses of subgroups and interactions, and sensitivity analyses | 4-5, Tab S2 |
| Discussion | | | |
| Key results | 18 | Summarise key results with reference to study objectives | 6 |
| Limitations | 19 | Discuss limitations of the study, taking into account sources of potential bias or imprecision. Discuss both direction and magnitude of any potential bias | 6-7 |
| Interpretation | 20 | Give a cautious overall interpretation of results considering objectives, limitations, multiplicity of analyses, results from similar studies, and other relevant evidence | 6-7 |
| Generalisability | 21 | Discuss the generalisability (external validity) of the study results | 6-7 |
| Other Information | | | |
| Funding | 22 | Give the source of funding and the role of the funders for the present study and, if applicable, for the original study on which the present article is based | 11 |

**Give information separately for cases and controls in case-control studies and, if applicable, for exposed and unexposed groups in cohort and cross-sectional studies.*

**Note:** An Explanation and Elaboration article discusses each checklist item and gives methodological background and published examples of transparent reporting. The STROBE checklist is best used in conjunction with this article (freely available on the Web sites of PLoS Medicine at http://www.plosmedicine.org/, Annals of Internal Medicine at http://www.annals.org/, and Epidemiology at http://www.epidem.com/). Information on the STROBE Initiative is available at www.strobe-statement.org.

## Table S2. Patient characteristics and distribution of confounding variables after propensity score matching. After matching, standardized differences of confounding variables were <0.1 except for brain irradiation (0.127) which was then included in the PSM model.

| Variables | Chemotherapy  N=99 (50%) | Checkpoint inhibitor  N=99 (50%) | P-value |
| --- | --- | --- | --- |
| Age (y), mean ± SD | 60.7 ± 9.4 | 61.0 ± 9.3 | 0.81 |
| Sex, female, n (%) | 40 (40%) | 37 (37%) | 0.66 |
| Smoking, n (%) |  |  | <0.001 |
| Never smoker | 1 (1%) | 3 (3%) |  |
| Smoker | 43 (43%) | 40 (40%) |  |
| Ex-smoker | 26 (26%) | 47 (47%) |  |
| n.a. | 20 (29 %) | 9 (9.1%) |  |
| Pack years, median (IQR) | 40 (30, 50) | 31 (20, 42) | 0.005 |
| Pathology, n (%) |  |  | 0.47 |
| SCLC | 94 (95%) | 95 (96%) |  |
| LCNEC | 5 (5%) | 3 (3%) |  |
| Other | 0 (0%) | 1 (1%) |  |
| Extensive disease, n (%) | 93 (94%) | 63 (64%) | <0.001 |
| Best response to 1L treatment, n (%) |  |  | <0.001 |
| CR | 1 (1%) | 3 (3%) |  |
| PR | 7 (7%) | 15 (15%) |  |
| SD | 32 (32%) | 9 (9%) |  |
| PD | 47 (47%) | 62 (63%) |  |
| n.a. | 12 (12 %) | 10 (10%) |  |
| Progression on 1L within 365 days, n (%) | 65 (65%) | 75 (75%) | 0.12 |
| Progression on 1L within 180 days, n (%) | 30 (30%) | 32 (32%) | 0.55 |
| ECOG at start of ≥2L, median (IQR) | 1 (0, 1) | 1 (1, 2) | 0.13 |
| Metastases at start of ≥2L, n (%) |  |  |  |
| Lung | 12 (12%) | 47 (47%) | <0.001 |
| Liver | 39 (39%) | 43 (43%) | 0.56 |
| Adrenal glands | 22 (22%) | 23 (23%) | 0.87 |
| Bone | 34 (34%) | 25 (25%) | 0.16 |
| Brain | 43 (43%) | 41 (41%) | 0.77 |
| Brain irradiation, n (%) | 68 (69%) | 62 (63%) | 0.37 |
| Abbreviations: BM, brain metastases; CPI, checkpoint inhibitor; CR, complete remission; CRT, cranial radiotherapy; ECOG, Eastern Cooperative Oncology Group; LCNEC, large cell neuroendocrine carcinoma; n.a., not available; PD, progressive disease; PR, partial remission; SCLC, small cell lung cancer; SD, stable disease; 1L, first line of therapy; ≥2L, second or further-line treatment. | | | |

## Table S3. Standardized differences in means of covariates used for propensity score matching. To adjust for imbalances, standardized mean differences (SMD) above 0.1 were included into the post-hoc regression model in the propensity score matched (PSM) cohort*.

| **Covariates** | **SMD before matching**  **(full cohort)**  **N=285** | **SMD after matching**  **(PSM cohort)**  **N=198** |
| --- | --- | --- |
| Age (quintiles) | 0.174 | 0.000 |
| Sex | 0.073 | 0.062 |
| Brain metastases | 0.022 | 0.042 |
| Liver metastases | 0.009 | 0.008 |
| Prior cranial radiotherapy (CRT) | 0.084 | 0.127* |
| Progressive disease within 180 days of first-line treatment | 0.085 | 0.043 |

## Table S4. Subgroup analysis of 79 patients where brain imaging was available within six weeks before treatment initiation.

| Variables | Chemotherapy  N=53 | Checkpoint inhibitor  N=26 | P-value |
| --- | --- | --- | --- |
| Brain metastases, n (%) | 52 (98%) | 16 (64%) | 0.002 |
| Prior whole brain irradiation, n (%) | 48 (91%) | 21 (81%) | 0.220 |
| Time to intracranial real-world progression, median (IQR) | 97 (71, 172) | 71 (31, 144) | 0.058 |
| 1-year intracranial real-world PFS | HR 1.49 (95% CI 0.78 to 2.86) | | 0.226 |
| Abbreviations: 95% CI, 95% confidence interval; HR, hazard ratio; IQR, interquartile range; PFS, progression-free survival. | | | |

# Supplementary Figures

## Figure S1. Univariate Kaplan-Meier survival analysis including a ‘number at risk’-table demonstrating 3-year overall survival among patients receiving second or further-line (≥2L) treatment with checkpoint inhibitors (CPI) versus chemotherapy (CTx) (p<0.001, logrank test).

## Figure S2. A scatter plot was used to assess the assumption of linearity between the outcome and continuous covariates (age). A histogram of age shows the age distribution in our cohort.
